# Timeliness of Childhood Vaccinations Following Strengthening of the Second Year of Life (2YL) Immunization Platform and Introduction of Catch-Up Vaccination Policy in Ghana

**DOI:** 10.3390/vaccines12070716

**Published:** 2024-06-27

**Authors:** Pierre Muhoza, Monica P. Shah, Kwame Amponsa-Achiano, Hongjiang Gao, Pamela Quaye, William Opare, Charlotte Okae, Philip-Neri Aboyinga, Joseph Kwadwo Larbi Opare, Daniel C. Ehlman, Melissa T. Wardle, Aaron S. Wallace

**Affiliations:** 1 Global Immunization Division, U.S. Centers for Disease Control and Prevention, Atlanta, GA 30329, USA; 2 Epidemic Intelligence Service, U.S. Centers for Disease Control and Prevention, Atlanta, GA 30329, USA; 3 Expanded Programme on Immunisation, Disease Control and Prevention Department, Public Health Division, Ghana Health Service, Accra P.O. Box M 44, Ghana; 4 Neglected Tropical Diseases Control Programme, Disease Control and Prevention Department, Public Health Division, Ghana Health Service, Accra P.O. Box M 44, Ghana

**Keywords:** immunization, life course, 2YL, catch-up vaccination, missed opportunities for vaccination (MOV), vaccination timeliness, age appropriate, quality improvement, Ghana, EPI

## Abstract

Strengthening routine immunization systems to successfully deliver childhood vaccines during the second year of life (2YL) is critical for vaccine-preventable disease control. In Ghana, the 18-month visit provides opportunities to deliver the second dose of the measles–rubella vaccine (MR2) and for healthcare workers to assess for and provide children with any missed vaccine doses. In 2016, the Ghana Health Service (GHS) revised its national immunization policies to include guidelines for catch-up vaccinations. This study assessed the change in the timely receipt of vaccinations per Ghana’s Expanded Program on Immunizations (EPI) schedule, an important indicator of service quality, following the introduction of the catch-up policy and implementation of a multifaceted intervention package. Vaccination coverage was assessed from household surveys conducted in the Greater Accra, Northern, and Volta regions for 392 and 931 children aged 24–35 months with documented immunization history in 2016 and 2020, respectively. Age at receipt of childhood vaccines was compared to the recommended age, as per the EPI schedule. Cumulative days under-vaccinated during the first 24 months of life for each recommended dose were assessed. Multivariable Cox regression was used to assess the associations between child and caregiver characteristics and time to MR2 vaccination. From 2016 to 2020, the proportion of children receiving all recommended doses on schedule generally improved, the duration of under-vaccination was shortened for most doses, and higher coverage rates were achieved at earlier ages for the MR series. More timely infant doses and caregiver awareness of the 2YL visit were positively associated with MR2 vaccination. Fostering a well-supported cadre of vaccinators, building community demand for 2YL vaccination, sustaining service utilization through strengthened defaulter tracking and caregiver-reminder systems, and creating a favorable policy environment that promotes vaccination over the life course are critical to improving the timeliness of childhood vaccinations.

## 1. Introduction

Endorsed by the World Health Assembly in 2020, the Immunization Agenda 2030 (IA2030) aims to reduce the morbidity and mortality caused by vaccine-preventable diseases (VPDs) worldwide [1]. The framework calls on countries to strengthen their immunization programs to reach at least 90% vaccination for all vaccines recommended in their national immunization schedule, including coverage with three doses of the diphtheria–tetanus–pertussis vaccine (DTP3) and two doses of the measles-containing vaccine (MCV2). DTP3 coverage by age 12 months indicates routine immunization (RI) program performance. In contrast, timely receipt of MCV2 is essential to prevent the spread of measles and is important for continued progress toward measles elimination goals [2].

In addition to high vaccination coverage, effective VPD control also requires the timely administration of recommended vaccines according to a specified national schedule [3]. Vaccination schedules aim to maximize the benefits of timely protection while minimizing VPD risk or adverse effects following immunization (AEFIs). Thus, schedules consider a range of age-specific factors, including susceptibility to disease and biological response to immunizations. Delayed vaccination increases susceptibility to VPDs, which can lead to increased morbidity and mortality [4,5,6]. Conversely, vaccine administration before the recommended age may attenuate vaccine effectiveness since maternal antibodies are still present in the child’s immune system and may interfere with the immune response to vaccination [7,8]. Thus, vaccination coverage and the timing of vaccination are both important metrics for evaluating the quality and effectiveness of vaccination programs.

The World Health Organization (WHO) recommends countries establish and strengthen a well-child visit in the second year of life (2YL), including vaccination and other services as part of a continuum of care [9]. In addition to providing vaccination opportunities for vaccine doses recommended during the 2YL, such as MCV2, a strengthened 2YL vaccination platform may also provide catch-up vaccination opportunities for children older than one year who are missing vaccine doses recommended during infancy [9].

In 2012, Ghana became the first country in the African region to introduce MCV2, during the 18-month visit, into its RI schedule [10,11]. At the time of MCV2 introduction, Ghana also introduced two infant vaccines, the pneumococcal conjugate vaccine (PCV) and the rotavirus vaccine, to be administered during established visits for pentavalent vaccine [12]. By 2015, coverage with the last doses of the PCV and rotavirus vaccines had reached 88%, levels similar to those of the more established third dose of the pentavalent vaccine, whereas MCV2 (offered as a combined measles–rubella [MR] vaccine since 2013 [13]) coverage lagged at 63%, underscoring challenges with vaccination beyond infancy [14].

From 2015 to 2020, the Ghana Health Service (GHS), in collaboration with the U.S. Centers for Disease Control and Prevention (CDC), designed and implemented interventions to strengthen the 2YL platform and to facilitate the introduction of the meningococcal A conjugate vaccine (Men A) in 2016. During early to mid-2016, the CDC and GHS conducted baseline health facility and household surveys in three underperforming regions of the country to understand the factors associated with poor MCV2 coverage [11]. The baseline assessment highlighted region-level inequities in coverage across various vaccine doses, with several factors contributing to low MCV2 coverage, including a 9-year gap, since the most recent EPI staff training; insufficient supportive supervision and defaulter tracing; weak communication between health care workers (HCWs) and caregivers; and poor HCW practices around documentation of immunization data coupled with inadequate stocks of updated immunization recording and reporting tools [11,15].

To address the gaps highlighted by the baseline assessment, a multifaceted package of 2YL strengthening strategies was designed and implemented in the selected regions. On the service-delivery side, interventions broadly included improving the quality and frequency of EPI training and supportive supervision to improve RI program performance at the district and health-facility levels. HCW capacity-building activities focused on several key areas, including strengthening defaulter tracing, providing catch-up vaccine doses, improving recording and reporting practices of catch-up and 2YL vaccine doses, reinforcing practices related to vaccine vial-opening policies, and enhancing interpersonal communication with caregivers about the importance of 2YL vaccination. Social mobilization and demand-generation activities had a broader scope, primarily centered on increasing vaccine confidence and awareness about the importance of 2YL vaccination among caregivers and their communities. These efforts included several approaches, such as door-to-door interpersonal communication, referring individuals for catch-up vaccinations, disseminating tailored messages for 2YL vaccination through mass-media channels, engaging with local community leaders, and collaborating with daycare proprietors to promote the benefits of immunization.

Additionally, these activities strived to improve screening and referral practices at daycare facilities. In 2016, EPI policies were revised to include guidelines around catch-up vaccination for children under the age of five years with missing vaccine doses. Immunization data-collection tools and databases were also revised to include indicators for 2YL vaccination.

Understanding the impact of the 2YL project activities on the timing of childhood vaccination may provide insights for refining strategies to enhance timely vaccine administration and child survival. In this study, we assessed the change in vaccination timeliness among children aged 24–35 months before and after project implementation from 2016 to 2020 in three regions of Ghana. We also evaluated the extent to which children were under-vaccinated for vaccine doses during the first 24 months of life. This study makes an important contribution to the limited understanding of vaccination timeliness in Ghana, since previous studies had a limited geographic scope, focused solely on infant vaccines, and were conducted prior to the establishment of the 2YL platform [16,17,18,19]. Although previous research suggested that delays in infant vaccinations were common in Ghana, similar to other low- and middle-income countries (LMIC), the extent of subnational variation in vaccination timeliness remains to be explored [20].

## 2. Materials and Methods

### 2.1. Study Site

The present study includes a subset of data collected as part of the broader 2YL initiative in the Greater Accra Region (GAR), Northern Region (NR), and Volta Region (VR) of Ghana [11]. Although Ghana reorganized its administrative regions in 2018 following a referendum vote to create new regions, this study used the pre-2018 regional boundaries for GAR, NR (now the Northern, North East, and Savannah Regions), and VR (now the Oti and Volta Regions) for both surveys to ensure comparability of findings. According to the 2021 national census, these regions collectively accounted for 37.2% of Ghana’s total population of 30.8 million inhabitants [21]. They were selected because they had lower-than-expected MR2 coverage due to various programmatic challenges. GAR is predominantly urban, characterized by higher levels of education, high-income variability, a dense population, and high residential mobility [22]. The population in VR, largely separated from the rest of Ghana by Lake Volta, the country’s largest lake, experiences barriers to access to healthcare, including RI services [23]. NR is predominantly rural, and while it is the country’s largest region, it has one of the lowest levels of socio-economic and educational attainment in Ghana. Additionally, it is sparsely populated and includes seasonally mobile pastoralist communities with infrequent contact with the health system.

### 2.2. Sampling and Participant Selection

This study was derived from baseline and endline household surveys conducted in March 2016 and August 2020 to estimate the immunization coverage among children 12–35 months old [11,24]. The details of the baseline and endline household survey design have been previously described elsewhere [11,24]. Briefly, both surveys used a stratified multi-stage cluster sample design. Within each region, enumeration areas (EAs) were selected by probability proportional to size, and, following a listing of all households in selected EAs, a simple random sample was drawn. In households with eligible children in the 12–23 month or 24–35 month age groups, caregivers could respond for up to two children (i.e., one interview per child in each age group). If multiple children were in the 12–23 month or 24–35 month age group, one child was chosen randomly. For the endline survey, children were eligible if they were aged 12–35 months as of March 2020. The selection of the cutoff was intended to minimize potential bias in the analytic sample related to the disruptions in immunization services caused by the COVID-19 pandemic. Trained field staff administered a standardized questionnaire to the selected child’s caregiver. The survey questions included demographics and characteristics of the child, caregiver, and household; the caregiver’s immunization awareness, knowledge, attitudes, beliefs, and perceptions regarding family, community, and healthcare worker support for immunizations; and the childhood vaccination history assessed by home-based vaccination record (child health record booklet—CHRB) data, health-facility records, or caregiver recall.

The present study reports on data collected from children ages 24 to 35 months with vaccination status and date of vaccination validated using either the child’s CHRB or health-facility records.

This project was approved by GHS’s Ethics Review Committee and the U.S. Centers for Disease Control and Prevention.

### 2.3. Definitions and Derived Variables

The age of the vaccination receipt was calculated in days for each recommended dose by subtracting the date of vaccination from the child’s date of birth. Vaccination timeliness was assessed by comparing the age of vaccination receipt with the age recommendations listed in Ghana’s immunization schedule (Table 1). Based on the age of vaccination receipt, a dose was considered timely if the vaccine was administered within 28 days after the recommended age. Early doses were those received before the earliest nationally accepted valid age. A dose was considered valid if received on or after the minimum recommended age. For multi-dose antigens, validity was determined if they were received on or after the minimum interval (i.e., 4 weeks). For each specific dose, days under-vaccinated were used as a continuous measure to assess the time in days between the end of each recommended age range until either when the child was vaccinated or when 24 months was reached. We considered each recommended age range to begin at the fewest number of days and to end at the greatest number of days possibly composing the given number of months (Table 1).

### 2.4. Data and Statistical Analysis

Among children aged 24–35 months with written documentation of their immunization history, we estimated age-specific coverage rates for doses in the MR series calculated as the cumulative percentage of children vaccinated up to that month. Percentage estimates and their 95% confidence intervals (CIs) accounting for survey design and region-specific sampling weights were calculated and displayed graphically. We reported the median number of days for each vaccine dose, with the interquartile range (IQR), during which children were under-vaccinated for the first 24 months of life. Medians and proportions were compared using the Mann–Whitney and chi-square tests, respectively.

To describe factors associated with the timeliness of 2YL vaccination, we conducted an exploratory analysis evaluating the influence of (1) caregiver awareness of the 18-month visit and (2) the number of valid and timely infant doses on time to MR2 vaccination adjusting for key demographic characteristics of the child (age, sex, birth order), mother (age, education, marital status, religion), the household (urban/rural settlement) and year of the survey. Caregiver knowledge of the immunization schedule is an established predictor of childhood vaccination, and improving caregiver awareness of the 2YL visit was a key objective of the intervention package, with elements addressing this issue at the community and health-facility levels. Given that previous studies suggested that delayed vaccinations during infancy may influence the likelihood of completing the immunization schedule recommended during the first year of life [25,26], we extrapolated from those findings to hypothesize timely receipt of infant vaccinations would be associated with timely 2YL vaccine receipt.

Factors associated with time to MR2 vaccination (defined as the number of days between the recommended vaccination age and the date MR2 was given) were examined using univariable and multivariable Cox regression and reported as crude and adjusted hazard ratio (HR and aHR), respectively, where HR < 1 implies delayed vaccination compared with the referent group (“hazard” being the conditional probability of receiving MR2 at time *t*, given that MR2 has not been received before time *t*). The hazard ratio compares the average instantaneous risk of vaccination with MR2 over the study period (the hazard) among a randomly chosen pair of children, one from the exposed group and another from the non-exposed group. Children with no MR2 vaccination recorded were right-censored at the age of the interview. We assessed the proportional hazards assumption for each covariate using graphical plots (log-negative log survival plots). The assumption of proportional hazards was violated for the child’s age. Thus, we computed age-stratified Cox regression models, allowing the baseline hazards to vary by the child’s age. These models were implemented separately for each region, with ‘hazard’ here on referred to as ‘likelihood’.

All analyses were performed using the appropriate clustering and weighting statements to account for the complex survey design described above. Given our analytic sample’s inclusion criteria, we used Stata’s *subpop* option to conduct analyses using the complete data file to maintain an accurate variance estimation. The Taylor series linearization method was used to calculate the variance of the parameter estimates. Unweighted case frequencies and weighted proportions are reported. All analyses were performed using Stata version 17 [27].

## 3. Results

### 3.1. Participant Characteristics

Table 2 describes the characteristics of the surveyed children and their households for each region. A total of 464 and 959 children aged 24–35 months across the three regions were surveyed for the 2016 and 2020 surveys, respectively. The present study included a subset of 392 and 931 children with vaccination records in 2016 and 2020, respectively. A detailed description of the analytic subsets is provided in Supplementary Figure S1. From 2016 to 2020, the vaccination record availability (primarily driven by CHRB availability) increased significantly across all regions (Table 2). In 2016, the vaccination record availability ranged from 76.9% (95% CI: 61.0, 87.7) in GAR to 89.3% (95% CI: 82.8, 93.6) in NR. In 2020, it ranged from 91.4% (95% CI: 86.5, 94.6) in GAR to 99.5% (95% CI: 97.8, 99.9) in VR.

The median age of the children with vaccination records was 29 months (IQR: 27, 33; Table 2). Nearly half of these children were male, with a higher proportion (69.1%) in NR in 2016 compared to 2020. The respondent was primarily the child’s mother (94.6% in GAR, 92.6% NR, and 89.9% in VR), and the median maternal age for both survey rounds was 30 years, ranging from 29.5 years in VR in 2016 to 32 in GAR in 2020. While the proportion of children from urban settlements was >92% for GAR in both survey rounds, there was temporal variation in NR (23.8–30.0%) and VR (30.2–26.7%). Most mothers were Christian in GAR and VR (≥88% for both years) and Muslim in NR (49.5% in 2016 and 72.2% in 2020). From 2016 to 2020, caregiver awareness of the need for an 18-month visit before the second birthday increased significantly in NR (19–46.7%) and VR (36.8–57.0%) but decreased in GAR (62.8–58.3%). Timely receipt of infant vaccinations also improved from 2016 to 2020 in NR and VR, but not in GAR, with an increase in the proportion of children who received more than 10 timely infant doses in NR (24.1–30.0%) and VR (49.3–59.6%), but a decrease in GAR (60.1–56.1%). Nonetheless, GAR experienced an improvement in the proportion of children receiving 6–10 timely doses (17.7–24.3%).

### 3.2. Timing of Vaccinations

**Figure 1:**
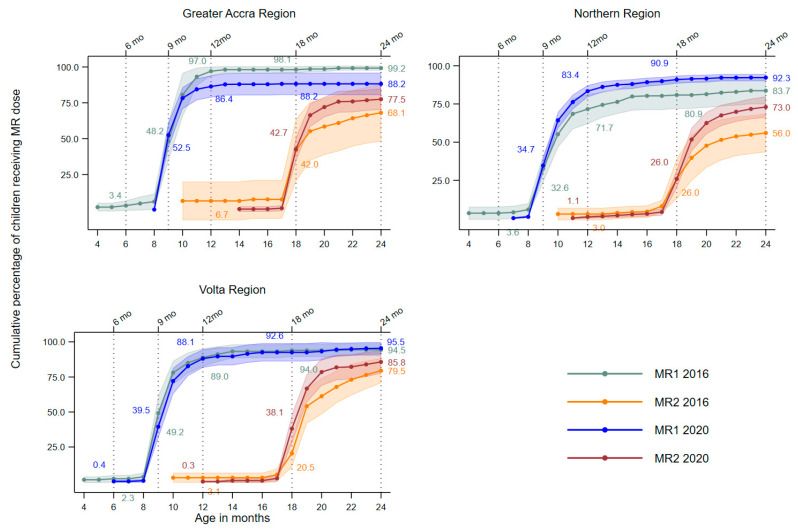
Cumulative vaccination coverage by age in months for the first and second dose of measles–rubella (MR) vaccine among children aged 24–35 months in Greater Accra, Northern, and Volta regions in 2016 and 2020. Values indicate cumulative percentage of children vaccinated by 6, 9, 12, 18, and 24 months of age. Shaded areas represent the 95% confidence intervals for the coverage estimates.

Figure 1 illustrates the cumulative percentage of children aged 24–35 months vaccinated with the MR series by region. From 2016 to 2020, the cumulative proportion of children who received MR1 by the recommended age of 9 months increased in GAR (48.2% to 52.5%) and NR (32.6% to 34.7%) but decreased in VR (49.2% to 39.5%). Over the same period, the cumulative proportion of children who received MR2 by the recommended age of 18 months increased in VR (20.5–38.1%) and remained unchanged in GAR (42%) and NR (26.0%).

Figure 1 also highlights the dropout from MR1 to MR2 by region between survey rounds, with a lower coverage of MR2 than MR1 at each survey. In all regions, some children were vaccinated as early as 4 months for MR1 and 10 months for MR2, albeit less frequently in 2020 when compared with 2016. The slopes of the curves around the recommended times of receipt for both doses were steep in 2020 compared to the more gradual slopes observed in 2016, which is indicative of higher coverage levels being achieved more rapidly in 2020 relative to 2016 across all regions. For example, between the ages of 9 and 12 months, MR1 coverage increased from 32.6% to 71.7% in 2016 and from 34.7% to 83.4% in 2020 among children in NR. In this cohort, MR1 coverage reached 83.7% and 92.3% by the end of 2YL during 2016 and 2020, respectively. Similarly, whereas MR2 coverage at 18 months was 26% for both years among children in NR, it increased to 56% in 2016 and to 73.0% in 2020 by 24 months.

In 2016, the recommended minimum coverage of 90% for MR1 was achieved by 11 months in GAR, by 13 months in VR, and not achieved in NR (Figure 1). In 2020, 90% MR1 coverage was achieved by 14 months in both NR and VR and not achieved in GAR. Although the recommended minimum coverage of 90% for MR2 was not achieved in any region by 24 months of age, catch-up vaccination during the 2YL enabled increases in coverage across all regions. VR was closest to achieving the coverage target in 2020, with cumulative MR2 coverage at 24 months reaching 85.8%.

### 3.3. Duration of Under-Vaccination during the First 24 Months of Life

Across the immunization series, the duration of under-vaccination tended to be longer for most doses in NR compared to other regions for both survey rounds (Table 3). From 2016 to 2020, the median number of days under-vaccinated decreased for most doses across all regions. Nonetheless, the results were mixed in GAR, with the duration of under-vaccination increasing significantly for some doses, including BCG, yellow fever (YF), and the oral poliovirus vaccine (OPV) series. Notably, the median duration of MR2 under-vaccination decreased significantly across all regions, by 68 days in NR and 52 days in both GAR and VR.

Across all regions and for both survey rounds, the duration of under-vaccination generally increased with each subsequent scheduled visit and tended to be similar for scheduled doses during the same visit (Table 3). The exception was the duration of under-vaccination for OPV doses in 2020, which tended to be longer relative to the doses scheduled during the same visit. For instance, the median number of days under-vaccinated with the third dose of OPV in GAR in 2020 was 46 but was only 16 for the third dose of the pentavalent vaccine.

From 2016 to 2020, the proportion of children aged 24–35 months receiving all recommended doses on a timely basis increased in NR (9.9% to 13.6%) and VR (24.6% to 27.3%) but decreased in GAR (33.6% to 30.0%). None of these changes were statistically significant.

### 3.4. Risk Factors for Time to MR2 Vaccination

In multivariable Cox regression models, several factors were significantly associated with time to MR2 vaccination (Table 4). Across all regions, an increased number of valid, timely vaccine doses during infancy was associated with an increased likelihood of MR2 vaccination. In NR, for example, as compared with children with no timely doses, those receiving 1–5, 6–10, and >10 timely doses, respectively, experienced a 1.9 (95% CI: 1.3, 2.6), 2.1 (95% CI: 1.6, 2.9), and 2.2 (95% CI: 1.6, 3.0) higher likelihood of MR2 vaccination. Caregiver’s awareness of the 18-month visit was significantly associated with an increased likelihood of MR2 vaccination in NR (aHR: 1.2, 95% CI: 1.0–1.5) and VR (aHR: 1.5, 95% CI: 1.2–1.8), although the association was not significant in GAR (aHR: 1.2, 95% CI: 0.9–1.6). In GAR and NR, but not VR, children of older mothers had a higher likelihood of MR2 vaccination when compared to those of mothers aged <25 years. Certain factors significantly positively impacted the likelihood of MR2 vaccination in some regions, but not others. These factors included earlier rank in birth order in NR and VR, male sex and rural residence in NR, and Christian religion in GAR and NR.

## 4. Discussion

This study highlights improvements in the timely receipt of childhood vaccinations, an important indicator of immunization service quality, following the implementation of an initiative to strengthen the 2YL vaccination platform and introduce catch-up vaccination policies in Ghana. From 2016 to 2020, under-vaccination occurred for fewer doses and shorter duration. The proportion of children vaccinated by the recommended age was higher in 2020 vs. 2016. The improvements were noted for vaccine doses recommended during infancy and those offered during 2YL. Although the improvements varied across doses and regions, the results suggest that, overall, more children were likely protected at earlier ages against VPDs following the implementation of the immunization system strengthening strategies. While the increases in coverage due to catch-up vaccination were marginal in some regions, at the population level, they represent meaningful benefits needed to stop disease transmission. For example, the recent increase in measles cases in Ghana following several years of low disease incidence implies persistent pockets of susceptible hosts sustaining transmission [13,28,29]. In addition to local measles spread, Ghana remains at high risk for case importation given the country’s proximity to countries lagging in measles control in an increasingly integrated region [30]. The improvements in vaccination timeliness are, therefore, critical, since immunity gaps resulting from vaccination delays or inadequate immune response to vaccination can be exploited by case importations resulting in disruptive outbreaks.

Vaccination-timing results for the MR series showed improvements in coverage during 2YL, illustrating the importance of strengthening the utilization of the 2YL platform to facilitate catch-up vaccination and, ultimately, sustain progress toward the IA2030 goals. Nonetheless, since the recommended minimum MR2 coverage of 90% was not attained among children aged 24–35 months in any region (consistent with national MR2 coverage of 83% reported in 2021 [31]), further opportunities remain for improvement in coverage. Screening vaccination status at the time of school entry and providing catch-up vaccinations for those with missed doses may be an additional effective strategy to reduce delays and improve coverage among these older cohorts. Ghana has attained high net pre-primary and primary school enrollment rates (~75% and >80%, respectively), particularly among children in urban localities, and has also achieved gender parity in enrollment [32,33]. This means that, for regions like NR, where access to schools has improved [34] and our analysis suggested urban–rural and gender disparities in time to MR2 vaccination, school-based screening and vaccination at school entry could be attractive strategies to provide equitable catch-up vaccination opportunities [35]. Urban–rural disparities in time to MR2 vaccination are especially noteworthy, since they are consistent with previous studies showing lower MR series coverage and higher measles and rubella incidence in Ghanaian urban areas [13,36,37].

Since preventing the administration of invalid vaccine doses is, to a greater degree, the responsibility of healthcare workers, the decrease in the administration of early vaccine doses over the study period points to the relative success of staff training and supervision efforts. According to the Ghana EPI field guide [38], doses administered before the recommended age (age invalid) or not following the minimum spacing intervals (interval invalid) should be readministered to ensure adequate protection. Thus, in addition to potentially reducing vaccine efficacy [39], early vaccination may also inadvertently lead to avoidable increases in programmatic costs and unnecessary risk of AEFIs [40]. Overall, our findings corroborate previous studies showing that age- and interval-invalid vaccine doses are common in African countries [41,42]. Further research is needed to understand better the underlying reasons for invalid dose administration and improve adherence to the EPI schedule.

Because the duration of under-vaccination tended to be similar for doses scheduled during the same visit suggests high levels of receipt of multiple vaccines during the same visit. Leveraging contacts with caregivers of children to provide multiple vaccinations during the same visit, including appropriate catch-up doses, is critical for minimizing missed opportunities for vaccination (MOVs), decreasing the risk of defaulting from the system, and reducing programmatic costs. Further, simultaneous vaccinations, where appropriate, may also reduce caregivers’ opportunity costs, such as time away from work, travel time, transportation costs, or the need for childcare while away. Our findings nonetheless point to challenges with the consistent provision of simultaneous vaccinations given that OPV, IPV, and MenA doses tended to have longer delays than the other scheduled doses recommended during the same visit. First, IPV and MenA were, respectively, introduced in Ghana in 2018 and 2016, and it is likely the implementation delays and inefficiencies associated with new vaccine introductions, coupled with a lack of awareness among HCWs and caregivers about the new doses, contributed to the observed vaccination delays. Previous studies evaluating IPV introduction have highlighted that HCWs and caregivers may have concerns about the pain and possible adverse effects of multiple injections, given that the vaccine was scheduled alongside several other more established injectable vaccines [43,44]. While not unique to IPV, such concerns may lead to deliberate delays or refusals [45]. This issue could be addressed by disseminating evidence-based vaccine safety and acceptability communication messages.

Additionally, implementing training and supervision programs focused on improving interpersonal communication between HCWs and caregivers can help address such concerns. Second, due to funding delays, Ghana experienced widespread stockouts for OPV and MenA in 2017 and 2019 [46], respectively, which likely meant many children were left unprotected for long periods. Stockouts adversely impact vaccination timeliness, since HCWs may be reluctant to open multi-dose vials for fewer children [47,48], leading to MOVs and frustration among caregivers who are turned away. This may contribute to hesitation about returning to complete the child’s scheduled vaccinations [49]. To increase health-system resiliency when stockouts or other disruptions occur, it is important to have systems in place enabling diligent follow-up and catch-up vaccination for children who have missed vaccines. This requires strengthening of defaulter tracking systems and a focus on strengthening record keeping to identify children with missing doses.

An increasing number of timely doses recommended during infancy was associated with an increased likelihood of MR2 receipt. This is consistent with previous observations that delayed vaccinations are an important predictor of not completing the first-year immunization schedule [25] and that children who complete their infant vaccines by their first year are more likely to receive 2YL vaccines [24]. Overall, the finding suggests that even early deviations from the recommended schedule may have persisting effects that adversely impact the timely receipt of 2YL vaccines, prolonging susceptibility to childhood VPDs. The deviations may reflect barriers to childhood immunization. Still, they may also result from intentional adjustments to the immunization schedule by HCWs to address delayed initiation of a multi-dose series or the re-administration of previous invalid doses while observing dose-spacing requirements. This highlights the importance of supporting HCWs and caregivers to prevent children from falling behind on immunizations and, as appropriate, to utilize 2YL contact points to catch up on missed doses.

Since its establishment in 1979, the Ghanaian EPI schedule has increased in complexity following various vaccine introductions over the years. The increased complexity has implications for vaccination timeliness, since previous studies have suggested assessing the vaccination status and scheduling age-appropriate catch-up regimens for children who have fallen behind on immunizations can be challenging for overburdened HCWs [50,51,52]. Quality training and supportive supervision, supplemented with simple job aids, may promote improvements in correct screening, recording of tools, and scheduling of doses. Mobile-based immunization decision support systems to automatically construct age-appropriate vaccination schedules for children have also shown promise in LMIC [53] and should be further explored in the Ghana setting. Importantly, field guides should also provide clear instructions and scenarios on what HCWs should do during encounters with children who are behind on immunizations, especially in the absence of written immunization records for the child.

Systematic reviews have shown caregiver reminder/recall systems are effective interventions for improving service utilization and vaccination timeliness [54,55]. These include a wide range of mediums ranging from person-to-person telephone calls, letters, home visits, and text messages to combinations used to remind caregivers that vaccinations are due (reminders) or overdue (recall). Emphasizing the reminder component of these systems would likely be more effective for encouraging timeliness and preventing a build-up of delayed doses, which may be more challenging to address later. Although reminders are effective in increasing vaccination timeliness [56], their impact is less understood in LMIC settings. Nonetheless, a randomized controlled trial in NR evaluating the effect of telephone-call reminders on timely neonatal vaccination showed a 10.5% increase in timely vaccination in the intervention arm compared to the control group [17]. Extending such research to examine the impact of phone-based reminders on vaccination timeliness during 2YL may better inform immunization programming.

In this study, caregiver awareness of the 18-month visit was positively associated with time to MR2 vaccination. The positive association suggests demand generation and social mobilization efforts may be important contributors to improving vaccine timeliness. Our findings suggest that targeted and proactive messaging to communities about the importance and timing of 2YL vaccines should be emphasized to improve vaccination timeliness. In areas like VR and NR, where religious affiliation is an important predictor of 2YL vaccination timeliness, involving local religious leaders and faith-based organizations in raising awareness about 2YL vaccinations and broader vaccine promotion activities may help mitigate community concerns and improve timely vaccine uptake. To incentivize utilization of the 2YL visit, programs may also consider integrated demand-creation strategies that raise awareness of the other preventative health services (e.g., deworming, growth monitoring, and bed net distribution) typically delivered jointly with 2YL immunizations. Lastly, high-quality respectful interpersonal communication skills are essential for HCWs to educate caregivers on the benefits of the 2YL visit and the importance of adherence to the recommended vaccination schedule. Previous research in Ghana and elsewhere has shown that rude HCW behavior, inattention to caregiver questions, and not receiving enough information during healthcare visits are major reasons caregivers do not bring back their children for vaccinations, especially when they fall behind [49,57,58,59,60].

## 5. Strengths and Limitations

The timeliness of childhood vaccinations has seldom been studied in Ghana, and our study is the first to assess the changes following the strengthening of the RI system using data from a household survey specifically designed to assess immunization coverage in the 2YL. We included data from multiple birth cohorts with high levels of vaccination record availability.

Nonetheless, the proportion of children with written documentation of vaccination status was significantly higher in 2020 compared to 2016 across all regions, potentially limiting the interpretation of the pre–post comparisons. CHRB prevalence has markedly improved in Ghana over recent decades [61], and it is possible the health-system improvements achieved by 2020 facilitated increased CHRB availability, such that our 2020 sample of children with CHRBs was more representative of the general population than our 2016 sample, particularly in terms of healthcare access and caregiver motivation to seek timely vaccinations. Given that caregiver awareness of the importance of the CHRB is associated with its retention and improved vaccination uptake [62,63], it is possible that the exclusion of children without vaccination records in our analyses potentially resulted in a sample of those with more knowledgeable and possibly more motivated caregivers in 2016 who were more likely to seek timely vaccinations. Overall, this sampling difference would have the effect of underestimating the true change in timeliness between 2016 and 2020. This is most evident in GAR, where MR1 coverage at 10 months and beyond was higher in 2016 compared with 2020, following significant increases in CHRB prevalence as well as maternal education levels, both of which are known to be correlated with childhood immunization outcomes.

Another limitation of the pre–post study design is the lack of control or comparison regions, which limits direct causal attribution of the observed changes to strengthening the 2YL platform. Thus, we cannot exclude the possibility the observed changes were attributable to contextual changes during the 4-year implementation period. Despite the potential for confounding from other efforts to improve immunization coverage, there were no other interventions in the study regions targeting 2YL vaccination platform strengthening during the study period. Future research should consider including control or comparison areas.

Additionally, the examined factors associated with time to 2YL vaccination were limited to those collected in the two survey rounds. There may be influential factors not captured by this analysis, such as health-system issues related to vaccine supplies or immunization program implementation, contextual barriers such as travel distance and poor infrastructure, and as previously mentioned, caregiver attitudes about vaccinations. Due to the cross-sectional nature of the data, causal inferences cannot be made. The study was designed and powered to make inferences at the regional level, and the results are not intended for national-level inferences. Nevertheless, our exploration of regional variations provides learning opportunities to tailor programs to increase access and utilization of the 2YL immunization services. Lastly, it is impossible to tease apart the relative effects of the different components of the 2YL intervention package. As such, it is unclear how much each component contributed to the observed changes in vaccination timeliness. In the context of limited resources for improving vaccination programs, additional research is needed to determine the relative impact and the cost–benefits of individual strategies and their efficient targeting. 

## 6. Conclusions

While the experience of strengthening the 2YL platform in Ghana was unique, the lessons learned nonetheless provide valuable information for other countries that are considering introducing 2YL vaccines or catch-up vaccination policies into their RI programs, particularly those with regions similar to our study sites. This study has shown substantial improvements in the timeliness of vaccinations in three regions in Ghana following the implementation of a package of interventions to strengthen the 2YL vaccination platform and the revision of the EPI policy to include catch-up vaccination guidelines. The results from this analysis demonstrate that improvements in the timeliness of vaccinations are achievable through comprehensive HCW capacity building, strategic community engagement initiatives, and an enabling policy environment encouraging vaccination during 2YL and beyond. Despite the general successes, regional and urban–rural disparities in vaccination timeliness were noted and will require targeted and multifaceted solutions to address. Additional efforts to not only improve vaccination coverage but to ensure that vaccines are received at their recommended schedule can strengthen the routine immunization platform.

## Figures and Tables

**Table 1:** Recommended and minimum ages for Ghana routine childhood immunization schedule.

Vaccine	Recommended Age of Administration	Age in Days When Dose Considered Early	Age in Days after Recommended Age When Under-Vaccination Count Initiated *
**BCG**	At birth or within 2 weeks of delivery	-	≥21 days
**OPV0**	At birth or within 2 weeks of delivery	-	≥21 days
**OPV1**	6 weeks (42 days)	<42 days	≥49 days
**PCV1**	6 weeks (42 days)	<42 days	≥49 days
**Pentavalent 1**	6 weeks (42 days)	<42 days	≥49 days
**Rotavirus 1**	6 weeks (42 days)	<42 days	≥49 days
**OPV2**	10 weeks (70 days)	<70 days	≥77 days
**PCV2**	10 weeks (70 days)	<70 days	≥77 days
**Pentavalent 2**	10 weeks (70 days)	<70 days	≥77 days
**Rotavirus 2**	10 weeks (70 days)	<70 days	≥77 days
**IPV**	14 weeks (98 days)	<98 days	≥105 days
**OPV3**	14 weeks (98 days)	<98 days	≥105 days
**PCV3**	14 weeks (98 days)	<98 days	≥105 days
**Pentavalent 3**	14 weeks (98 days)	<98 days	≥105 days
**MR1**	9 months (273 days)	<273 days	≥303 days
**Yellow Fever**	9 months (273 days)	<273 days	≥303 days
**Men A**	18 months (542 days)	<542 days	≥572 days
**MR2**	18 months (542 days)	<542 days	≥572 days

BCG, bacillus Calmette-Guérin; IPV, inactivated polio vaccine; Men A, meningitis serogroup A conjugate vaccine; MR, measles–rubella vaccine, OPV, oral polio vaccine; Pentavalent vaccine includes diphtheria, pertussis, tetanus, *Haemophilus influenzae* type b, and hepatitis B vaccines; PCV, pneumococcal conjugate vaccine. * Each recommended age range (either week or month of visit) is assumed to end at the greatest number of days that could compose the given number of weeks or months. Since BCG and OPV0 are recommended at birth, they can either be timely or delayed and cannot be administered too early unlike the other tracer vaccines.

**Table 2:** Characteristics of children 24–35 months of age in Greater Accra, Northern, and Volta regions, 2016–2020.

	Greater Accra				Northern				Volta			
	2016 N = 100		2020 N = 224		2016 N = 211		2020 N = 535		2016 N = 153		2020 N = 200	
	**n**	**% (95% CIs)**	**n**	**% (95% CIs)**	**n**	**% (95% CIs)**	**n**	**% (95% CIs)**	**n**	**% (95% CIs)**	**n**	**% (95% CIs)**
Vaccination record availability *												
Children with vaccination cards	**73**	**76.9 (61.0–87.7)**	**190**	**83.1 (75.9–88.5)**	**189**	**89.3 (82.8–93.6)**	**518**	**96.2 (94.2–97.5)**	**130**	**87.5 (80.1–92.3)**	**193**	**96.8 (89.2–99.1)**
Children with facility register verification	**0**	**0.0**	**15**	**8.3 (4.1–16.0)**	**0**	**0.0**	**10**	**2.2 (1.2–4.1)**	**0**	**0.0**	**5**	**2.6 (0.6–11.1)**
Children with either vaccination record	**73**	**76.9 (61.0–87.7)**	**205**	**91.4 (86.5–94.6)**	**189**	**89.3 (82.8–93.6)**	**528**	**98.4 (96.6–99.2)**	**130**	**87.5 (80.1–92.3)**	**198**	**99.5 (97.8–99.9)**
**Child’s sex**												
Female	37	45.8 (35.0–57.0)	98	49.8 (41.0–58.6)	**75**	**30.9 (23.2–39.8)**	**251**	**47.2 (42.8–51.8)**	66	48.9 (40.1–57.7)	105	54.7 (46.5–62.7)
Male	36	54.2 (43.0–65.0)	107	50.2 (41.4–59.0)	**114**	**69.1 (60.2–76.8)**	**277**	**52.8 (48.2–57.2)**	64	51.1 (42.3–59.9)	93	45.3 (37.3–53.5)
**Birth order**												
First	26	47.2 (28.4–66.9)	72	38.6 (30.9–46.9)	41	18.9 (13.8–25.2)	108	22.0 (17.9–26.8)	**30**	**22.2 (16.1–29.8)**	**63**	**34.8 (27.8–42.7)**
Second	20	18.1 (9.7–31.1)	54	24.6 (18.6–31.7)	39	20.1 (13.5–28.9)	122	23.9 (20.3–27.9)	**31**	**27.0 (20.8–34.3)**	**36**	**19.6 (13.6–27.4)**
Third or more	27	34.7 (20.8–51.7)	79	36.8 (30.9–43.2)	109	61.0 (50.4–70.7)	298	54.1 (48.4–59.7)	**69**	**50.8 (43.7–57.9)**	**99**	**45.6 (39.9–51.4)**
**Maternal age (years)**												
<25	10	13.8 (5.6–30.0)	20	9.0 (5.1–15.5)	**37**	**15.0 (8.6–24.9)**	**63**	**12.7 (9.4–16.9)**	29	23.3 (16.9–31.1)	44	23.4 (18.6–28.8)
25–34	33	46.5 (29.4–64.4)	106	50.8 (43.3–58.2)	**84**	**53.7 (38.6–68.2)**	**137**	**26.6 (22.2–31.6)**	55	40.5 (31.8–50.0)	80	41.2 (33.8–49.0)
≥35	30	39.7 (24.3–57.5)	79	40.2 (33.8–47.0)	**68**	**31.3 (21.2–43.4)**	**328**	**60.7 (54.4–66.6)**	46	36.2 (27.8–45.6)	74	35.5 (29.7–41.7)
**Maternal education**												
Never attended school	**10**	**9.6 (4.3–20.1)**	**15**	**6.9 (4.1–11.2)**	136	74.3 (64.0–82.4)	373	68.1 (60.5–74.8)	40	29.6 (18.8–43.4)	36	17.1 (9.8–28.0)
Primary	**15**	**30.3 (18.9–44.8)**	**16**	**6.6 (4.1–10.4)**	14	5.9 (2.9–11.4)	43	8.0 (5.4–11.6)	23	15.5 (10.1–23.0)	34	15.7 (10.5–23.0)
Secondary or higher	**47**	**60.1 (46.9–72.0)**	**174**	**86.5 (80.4–91.0)**	39	19.9 (13.9–27.6)	110	24.0 (18.1–31.1)	67	54.9 (40.8–68.3)	127	67.2 (53.2–78.6)
**Maternal marital status**												
Single/Divorced/Separated/Widowed	**4**	**2.8 (1.0–7.9)**	**27**	**14.2 (9.1–21.4)**	3	1.3 (0.4–4.1)	12	2.8 (1.1–6.6)	6	5.0 (2.1–11.4)	22	9.8 (6.2–15.1)
Married/Co-habitating	**69**	**97.2 (92.1–99.0)**	**178**	**85.8 (78.6–90.9)**	186	98.7 (95.9–99.6)	516	97.2 (93.4–98.9)	124	95.0 (88.6–97.9)	176	90.2 (84.9–93.8)
**Maternal religion**												
Christian	64	88.3 (77.6–94.3)	175	88.2 (79.2–93.6)	**66**	**34.9 (23.4–48.4)**	**125**	**22.1 (13.6–33.6)**	116	88.0 (76.9–94.2)	172	87.8 (79.7–92.9)
Muslim	7	8.1 (3.1–19.3)	29	11.0 (5.7–20.3)	**104**	**49.5 (32.6–66.5)**	**359**	**72.2 (59.2–82.3)**	5	4.2 (1.1–14.8)	13	5.6 (2.4–12.7)
Traditionalist	2	3.6 (0.9–13.4)	0	0.0 -4.4	**15**	**13.1 (4.2–33.9)**	**37**	**2.5 (1.8–10.1)**	9	7.8 (3.4–17.0)	5	2.9 (0.8–7.7)
None / Do not know	0	0.0–2.6	1	0.7 (0.1–4.8)	**4**	**0.0 (0.8–8.6)**	**7**	**1.3 (0.4–4.0)**	0	0.7 (0.1–5.2)	8	4.1 (1.8–9.1)
**Settlement type**												
Urban	65	92.2 (71.4–98.2)	183	93.0 (80.6–97.7)	64	23.8 (11.7–42.5)	145	30.0 (16.7–48.0)	45	30.2 (16.0–49.6)	54	26.7 (13.9–45.1)
Rural	8	7.8 (1.8–28.6)	22	7.0 (2.3–19.4)	125	76.2 (57.5–88.3)	383	70.0 (52.0–83.3)	85	69.8 (50.4–84.0)	144	73.3 (54.9–86.1)
**Caregiver aware of need for 18 mo visit before 2nd birthday**												
No	35	37.2 (23.0–54.0)	94	41.7 (31.4–52.9)	**154**	**81.0 (67.5–89.8)**	**278**	**53.3 (43.3–62.9)**	**78**	**63.2 (54.8–70.8)**	**88**	**43.0 (35.3–50.9)**
Yes	38	62.8 (46.0–77.0)	111	58.3 (47.1–68.6)	**35**	**19.0 (10.2–32.5)**	**250**	**46.7 (37.1–56.7)**	**52**	**36.8 (29.2–45.2)**	**110**	**57.0 (49.1–64.7)**
**Number of valid timely infant doses ^†^**												
None timely	**3**	**2.5 (0.8–7.2)**	**18**	**11.1 (6.2–18.9)**	36	16.2 (9.1–27.1)	82	14.6 (10.5–20.0)	9	6.8 (3.6–12.8)	19	7.5 (3.6–15.1)
1–5 doses	**10**	**19.7 (9.2–37.4)**	**20**	**8.6 (5.4–13.4)**	61	36.4 (27.6–46.2)	150	27.4 (21.9–33.7)	29	20.6 (14.4–28.6)	34	15.7 (11.6–21.1)
6–10 doses	**13**	**17.7 (9.2–31.2)**	**46**	**24.3 (18.1–31.8)**	45	23.3 (17.8–30.0)	149	28.0 (23.7–32.7)	29	23.2 (16.4–31.9)	33	17.2 (11.7–24.4)
>10 doses	**47**	**60.1 (42.2–75.7)**	**121**	**56.1 (48.6–63.2)**	47	24.1 (14.3–37.6)	147	30.0 (22.7–38.4)	63	49.3 (40.5–58.1)	112	59.6 (49.2–69.1)

* Source vaccination records include child health record books and facility-based registers. Total Ns shown and denominator of proportion includes all sampled children in the region during the specified survey round. Characteristics included in this table are among children with vaccination records. ^†^ Includes 14 doses total: bacillus Calmette-Guérin (BCG); pentavalent 1, 2, and 3; rotavirus 1, and 2; oral polio vaccine 1, 2, and 3; pneumococcal conjugate vaccine (PCV) 1, 2, and 3; yellow fever, measles–rubella 1. Sample sizes are unweighted, and percentages and 95% CIs are weighted. Bold values denote statistical significance of the design-based chi-square test comparing 2016 to 2020 at the *p* < 0.05 level.

**Table 3:** Days under-vaccinated with various doses of vaccine during the first 24 months among children aged 24–35 months * in Greater Accra, Northern, and Volta Regions, 2016 and 2020.

	Greater Accra				Northern				Volta			
	2016 (N = 71)		2020 (N = 205)		2016 (N = 189)		2020 (N = 528)		2016 (N = 128)		2020 (N = 198)	
Vaccine	n	Median (IQR)	n	Median (IQR)	n	Median (IQR)	n	Median (IQR)	n	Median (IQR)	n	Median (IQR)
BCG	**62**	**2 (1, 6)**	**177**	**4 (1, 19)**	168	22 (8, 65)	506	17 (6, 40)	112	10 (3, 24)	164	7 (2, 51)
OPV1	**21**	**16 (11, 54)**	**77**	**29 (11, 90)**	124	16 (10, 74)	393	24 (8, 62)	80	14 (7, 25)	113	13 (5, 30)
PCV1	21	18 (11, 96)	56	14 (7, 682)	122	18 (10, 90)	356	16 (6, 34)	83	14 (7, 27)	108	10 (5, 22)
Pentavalent 1	22	16 (11, 96)	57	14 (7, 682)	123	16 (10, 55)	358	17 (6, 34)	81	15 (7, 29)	110	10 (5, 23)
Rota1	20	16 (10, 54)	60	15 (7, 682)	127	17 (10, 77)	357	17 (6, 35)	80	14 (7, 27)	108	10 (5, 22)
OPV2	**42**	**10 (4, 41)**	**125**	**29 (7, 75)**	**159**	**30 (14, 130)**	**459**	**41 (14, 96)**	100	18 (8, 37)	138	14 (6, 38)
PCV2	42	19 (4, 41)	104	14 (5, 40)	**159**	**32 (14, 130)**	**438**	**23 (9, 48)**	99	18 (8, 37)	136	11 (6, 30)
Pentavalent 2	41	10 (4, 41)	105	12 (4, 40)	162	29 (14, 124)	440	24 (9, 49)	101	18 (8, 36)	138	12 (6, 38)
Rota2	42	19 (6, 41)	107	12 (4, 62)	**160**	**33 (14, 146)**	**439**	**24 (10, 52)**	99	18 (8, 36)	136	12 (6, 30)
OPV3	**50**	**16 (4, 47)**	**149**	**46 (12, 169)**	173	47 (23, 220)	493	59 (22, 356)	117	21 (9, 47)	161	18 (7, 57)
PCV3	52	17 (4, 46)	128	16 (5, 62)	**169**	**57 (24, 233)**	**484**	**33 (14, 65)**	116	21 (9, 47)	158	14 (6, 36)
Pentavalent 3	50	18 (4, 47)	130	16 (5, 42)	**171**	**47 (23, 175)**	**484**	**33 (14, 67)**	118	23 (9, 47)	162	15 (7, 43)
MR1	25	27 (15, 34)	58	54 (12, 428)	**99**	**97 (30, 428)**	**263**	**42 (16, 155)**	42	47 (13, 121)	87	33 (13, 135)
YF	**25**	**27 (15, 31)**	**73**	**58 (12, 428)**	99	126 (31, 428)	305	60 (24, 212)	40	47 (13, 122)	90	41 (19, 130)
MR2	**44**	**159 (71, 159)**	**100**	**107 (18, 159)**	**142**	**159 (61, 159)**	**335**	**91 (22, 159)**	**85**	**95 (20, 159)**	**95**	**43 (17, 159)**

* Cumulative days under-vaccinated during the first 24 months of life for a recommended vaccine. Under-vaccination begins after the end of a recommendation period and continues until the child is vaccinated or reaches 24 months of age. Sample includes only children with documented vaccination records. Inactivated polio vaccine and meningitis serogroup A conjugate vaccine were introduced in 2018 and 2016, respectively, and are excluded from this analysis. N represents total number of children under-vaccinated with any dose. Bold values denote statistical significance of the Mann–Whitney Wilcoxon test at the *p* < 0.05 level. BCG, bacillus Calmette-Guérin; MR, measles–rubella vaccine, OPV, oral polio vaccine; Pentavalent vaccine includes diphtheria, pertussis, tetanus, *Haemophilus influenzae* type b, and hepatitis B vaccines; PCV, pneumococcal conjugate vaccine; Rota, rotavirus vaccine.

**Table 4:** Factors associated with time to vaccination for second dose of measles–rubella (MR2) vaccine among children aged 24–35 months in Greater Accra, Northern, and Volta regions.

	Greater Accra				Northern				Volta			
	Unadjusted		Adjusted		Unadjusted		Adjusted		Unadjusted		Adjusted	
	HR	95% CI	HR	95% CI	HR	95% CI	HR	95% CI	HR	95% CI	HR	95% CI
**Child’s sex**												
Female	REF		REF		REF		REF		REF		REF	
Male	1.1	(0.8–1.4)	1.2	(0.9–1.6)	0.7	(0.6–0.9) **	0.7	(0.6–0.9) **	1.0	(0.9–1.2)	0.9	(0.8–1.2)
**Birth order**												
First	REF		REF		REF		REF		REF		REF	
Second	0.9	(0.6–1.4)	1.0	(0.7–1.5)	1.0	(0.7–1.5)	1.0	(0.7–1.3)	0.9	(0.6–1.3)	1.0	(0.8–1.4)
Third or more	0.8	(0.5–1.2)	0.8	(0.5–1.1)	1.0	(0.7–1.3)	0.8	(0.6–1.0) *	0.6	(0.5–0.8) *	0.7	(0.6–1.0) *
**Maternal education**												
Never attended school	REF		REF		REF		REF		REF		REF	
Primary	0.8	(0.4–1.8)	0.8	(0.4–1.6)	1.1	(0.8–1.6)	1.0	(0.7–1.4)	1.1	(0.6–1.8)	0.9	(0.6–1.6)
Secondary or higher	1.0	(0.6–1.7)	1.2	(0.8–1.8)	0.9	(0.7–1.2)	1.0	(0.8–1.3)	1.8	(1.2–2.7)	1.4	(0.9–2.2)
**Maternal age (Years)**												
<25	REF		REF		REF		REF		REF		REF	
25–34	1.9	(1.1–3.1) *	1.8	(1.0–3.3)	1.2	(0.9–1.6)	1.3	(1.0–1.6)	0.8	(0.5–1.0)	0.9	(0.6–1.3)
≥35	1.8	(1.1–3.0) *	2.1	(1.1–3.9) *	1.3	(1.0–1.7) *	1.5	(1.2–1.9) *	0.8	(0.6–1.1)	1.0	(0.7–1.4)
**Maternal marital status**												
Single/Divorced /Widowed	REF		REF		REF		REF		REF		REF	
Married/Co-habitating	1.0	(0.6–1.6)	0.8	(0.6–1.2)	1.2	(0.8–2.0)	1.0	(0.6–1.5)	1.2	(0.8–1.7)	1.3	(0.9–1.7)
**Maternal religion**												
Other	REF		REF		REF		REF		REF		REF	
Christian	1.2	(0.8–2.0)	1.4	(1.0–2.0) *	1.3	(0.9–1.8)	1.4	(1.0–1.9) *	1.6	(1.1–2.5) *	1.4	(0.9–2.1)
**Settlement type**												
Urban	REF		REF		REF		REF		REF		REF	
Rural	1.1	(0.6–2.1)	1.2	(0.8–1.7)	1.7	(1.3–2.3) **	2.0	(1.4–2.7) ***	1.3	(0.8–2.0)	1.1	(0.8–1.6)
**Caregiver aware of need for 18-month visit before second birthday**												
No	REF		REF		REF		REF		REF		REF	
Yes	1.8	(1.2–2.7) **	1.2	(0.9–1.6)	1.5	(1.2–2.0) **	1.2	(1.0–1.5) *	1.7	(1.4–2.2) ***	1.5	(1.2–1.8) ***
**Number of valid timely doses †**												
None timely	REF		REF		REF		REF		REF		REF	
1–5 doses	16.3	(4.3–62.3) ***	20.4	(6.3–65.8) ***	1.9	(1.3–2.6) ***	1.9	(1.3–2.6) ***	4.6	(2.2–9.4) ***	4.2	(2.0–8.9) ***
6–10 doses	25.5	(8.9–72.9) ***	31.3	(12.1–81.2) ***	2.1	(1.6–2.9) ***	2.5	(1.9–3.4) ***	6.4	(3.4–12.1) ***	6.2	(3.1–12.4) ***
>10 doses	31.1	(11.6–83.0) ***	36.4	(14.7–90.0) ***	2.2	(1.6–3.0) ***	3.2	(2.3–4.3) ***	7.1	(3.6–13.8) ***	6.1	(3.0–12.4) ***
**Year**												
2016	REF		REF		REF		REF		REF			REF
2020	1.1	(0.7–1.8)	0.8	(0.6–1.2)	1.3	(0.9–1.8)	0.9	(0.7–1.3)	1.0	(0.7–1.3)	0.7	(0.5–1.0) *

† Includes 14 doses total: bacillus Calmette-Guérin (BCG); pentavalent 1, 2, and 3; rotavirus 1, and 2; oral polio vaccine (OPV) 1, 2 and 3; pneumococcal conjugate vaccine (PCV) 1, 2, and 3; yellow fever vaccine, measles–rubella 1. * *p*-value < 0.05. ** *p*-value < 0.01. *** *p*-value < 0.001

## Data Availability

The datasets used and/or analyzed during the current project may be available upon reasonable request.

## Supplementary Materials

The following supporting information can be downloaded at https://www.mdpi.com/article/10.3390/vaccines12070716/s1: Figure S1: Flowchart of children included in vaccination timeliness analyses in Greater Accra (GAR), Northern (NR), and Volta (VR) regions during 2016 and 2020.

## References

[1] World Health Organization (2020). Immunization Agenda 2030: A Global Strategy to Leave No One Behind.

[2] Regional Committee for Africa (2011). Measles Elimination by 2020: A Strategy for the African Region.

[3] Dombkowski K.J., Lantz P.M., Freed G.L. (2002). The need for surveillance of delay in age-appropriate immunization. Am. J. Prev. Med..

[4] Grant C.C., Roberts M., Scragg R., Stewart J., Lennon D., Kivell D., Ford R., Menzies R. (2003). Delayed immunisation and risk of pertussis in infants: Unmatched case-control study. BMJ.

[5] Kolos V., Menzies R., McIntyre P. (2007). Higher pertussis hospitalization rates in indigenous Australian infants, and delayed vaccination. Vaccine.

[6] Inskip H.M., Hall A.J., Chotard J., Loik F., Whittle H. (1991). Hepatitis B vaccine in the Gambian Expanded Programme on Immunization: Factors influencing antibody response. Int. J. Epidemiol..

[7] Sato H., Albrecht P., Reynolds D.W., Stagno S., Ennis F.A. (1979). Transfer of measles, mumps, and rubella antibodies from mother to infant: Its effect on measles, mumps, and rubella immunization. Am. J. Dis. Child..

[8] Lochlainn L.M.N., de Gier B., van der Maas N., van Binnendijk R., Strebel P.M., Goodman T., de Melker H.E., Moss W.J., Hahné S.J.M. (2019). Effect of measles vaccination in infants younger than 9 months on the immune response to subsequent measles vaccine doses: A systematic review and meta-analysis. Lancet Infect. Dis..

[9] World Health Organization (2018). Establishing and Strengthening Immunization in the Second Year of Life: Practices for Vaccination Beyond Infancy.

[10] Masresha B.G., Luce R., Okeibunor J., Shibeshi M.E., Kamadjeu R., Fall A. (2018). Introduction of the Second Dose of Measles Containing Vaccine in the Childhood Vaccination Programs Within the WHO Africa Region—Lessons Learnt. J. Immunol. Sci..

[11] Nyaku M., Wardle M., Eng J.V., Ametewee L., Bonsu G., Opare J.K.L., Conklin L. (2017). Immunization delivery in the second year of life in Ghana: The need for a multifaceted approach. Pan Afr. Med. J..

[12] Le Gargasson J.-B., Nyonator F.K., Adibo M., Gessner B.D., Colombini A. (2015). Costs of routine immunization and the introduction of new and underutilized vaccines in Ghana. Vaccine.

[13] Dongdem A.Z., Alhassan E., Opare D., Boateng G., Bonsu G., Amponsa-Achiano K., Sarkodie B., Dzotsi E., Adjabeng M., Afagbedzi S. (2021). An 11-year trend of rubella incidence cases reported in the measles case-based surveillance system, Ghana. Pan Afr. Med. J..

[14] World Health Organization & UNICEF (2022). Ghana: WHO and UNICEF Estimates of Immunization Coverage: 2021 Revision. https://data.unicef.org/wp-content/uploads/cp/immunisation/gha.pdf.

[15] Tchoualeu D.D., Harvey B., Nyaku M., Opare J., Traicoff D., Bonsu G., Quaye P., Sandhu H.S. (2021). Evaluation of the impact of immunization second year of life training interventions on health care workers in Ghana. Glob. Health Sci. Pract..

[16] Laryea D.O., Abbeyquaye Parbie E., Frimpong E. (2014). Timeliness of childhood vaccine uptake among children attending a tertiary health service facility-based immunisation clinic in Ghana. BMC Public Health.

[17] Levine G., Salifu A., Mohammed I., Fink G. (2021). Mobile nudges and financial incentives to improve coverage of timely neonatal vaccination in rural areas (GEVaP trial): A 3-armed cluster randomized controlled trial in Northern Ghana. PLoS ONE.

[18] Gram L., Soremekun S., ten Asbroek A., Manu A., O’Leary M., Hill Z., Danso S., Amenga-Etego S., Owusu-Agyei S., Kirkwood B.R. (2014). Socio-economic determinants and inequities in coverage and timeliness of early childhood immunisation in rural G hana. Trop. Med. Int. Health.

[19] Akmatov M.K., Mikolajczyk R.T. (2012). Timeliness of childhood vaccinations in 31 low and middle-income countries. J. Epidemiol. Community Health.

[20] Clark A., Sanderson C. (2009). Timing of children’s vaccinations in 45 low-income and middle-income countries: An analysis of survey data. Lancet.

[21] Ghana Statistical Service (2021). Ghana 2021 Population and Housing Census, Volume 1. https://census2021.statsghana.gov.gh/gssmain/fileUpload/reportthemelist/PRINT_COPY_VERSION_FOUR%2022ND_SEPT_AT_8_30AM.pdf.

[22] Banchani E., Tenkorang E.Y., Midodzi W. (2022). Examining the effects of individual and neighbourhood socio-economic status/wealth on hypertension among women in the Greater Accra Region of Ghana. Health Soc. Care Community.

[23] Sheff M.C., Bawah A.A., Asuming P.O., Kyei P., Kushitor M., Phillips J.F., Kachur S.P. (2020). Evaluating health service coverage in Ghana’s Volta region using a modified Tanahashi model. Glob. Health Action.

[24] Muhoza P., Shah M.P., Gao H., Amponsa-Achiano K., Quaye P., Opare W., Okae C., Aboyinga P.-N., Opare K.L., Wardle M.T. (2023). Predictors for Uptake of Vaccines Offered during the Second Year of Life: Second Dose of Measles-Containing Vaccine and Meningococcal Serogroup A-Containing Vaccine, Ghana, 2020. Vaccines.

[25] Janusz C.B., Frye M., Mutua M.K., Wagner A.L., Banerjee M., Boulton M.L. (2021). Vaccine Delay and Its Association With Under-vaccination in Children in Sub-Saharan Africa. Am. J. Prev. Med..

[26] Emmanuel O.W., Samuel A.A., Helen K.L. (2015). Determinants of childhood vaccination completion at a peri-urban hospital in Kenya, December 2013-January 2014: A case control study. Pan Afr. Med. J..

[27] StataCorp (2021). Stata Statistical Software: Release 17.

[28] Sheriff A.A., Zakariah A., Dapaa S., Odikro M.A., Issahaku R.G., Bandoh D., Noora C.L., Gebru G.N., Kenu E. (2023). Ghana’s progress towards measles elimination: Surveillance data analysis, Greater Accra Region, 2015–2019. Front. Trop. Dis..

[29] World Health Organization (2023). Provisional Monthly Measles and Rubella Data: Distribution of Measles Cases by Country and by Month, 2011–2023. https://www.who.int/teams/immunization-vaccines-and-biologicals/immunization-analysis-and-insights/surveillance/monitoring/provisional-monthly-measles-and-rubella-data.

[30] Wariri O., Nkereuwem E., Erondu N.A., Edem B., Nkereuwem O.O., Idoko O.T., Agogo E., Enegela J.E., Sesay T., Conde I.S. (2021). A scorecard of progress towards measles elimination in 15 west African countries, 2001–2019: A retrospective, multicountry analysis of national immunisation coverage and surveillance data. Lancet Glob. Health.

[31] Masresha B., Hatcher C., Lebo E., Tanifum P., Bwaka A., Minta A., Antoni S., Grant G.B., Perry R.T., O’Connor P. (2023). Progress toward measles elimination—African Region, 2017–2021. Morb. Mortal. Wkly. Rep..

[32] UNESCO Institute for Statistics Education and Literacy—Participation in Education 2023. https://uis.unesco.org/en/country/gh.

[33] Education Policy and Data Center (FHI 360) (2018). Ghana National Education Profile 2018 Update. https://www.epdc.org/sites/default/files/documents/EPDC_NEP_2018_Ghana.pdf.

[34] Akyeampong K., Djangmah J., Oduro A., Seidu A., Hunt F. Access to Basic Education in Ghana: The Evidence and the Issues; Country Analytic Report; ERIC: 2007. https://files.eric.ed.gov/fulltext/ED508809.pdf.

[35] Vandelaer J., Olaniran M. (2015). Using a school-based approach to deliver immunization—Global update. Vaccine.

[36] Asuman D., Ackah C.G., Enemark U. (2018). Inequalities in child immunization coverage in Ghana: Evidence from a decomposition analysis. Health Econ. Rev..

[37] Moran E.B., Wagner A.L., Asiedu-Bekoe F., Abdul-Karim A., Schroeder L.F., Boulton M.L. (2020). Socio-economic characteristics associated with the introduction of new vaccines and full childhood vaccination in Ghana, 2014. Vaccine.

[38] Ghana Health Service (GHS) (2016). Field Guide for the Ghana Immunization Programme.

[39] World Health Organization (2019). Measles vaccines: WHO position paper, April 2017—Recommendations. Vaccine.

[40] Feikema S.M., Klevens R.M., Washington M.L., Barker L. (2000). Extraimmunization among US children. JAMA.

[41] Akmatov M.K., Kimani-Murage E., Pessler F., Guzman C.A., Krause G., Kreienbrock L., Mikolajczyk R.T. (2015). Evaluation of invalid vaccine doses in 31 countries of the WHO African Region. Vaccine.

[42] Tsega A., Hausi H., Chriwa G., Steinglass R., Smith D., Valle M. (2016). Vaccination coverage and timely vaccination with valid doses in Malawi. Vaccine Rep..

[43] Dolan S.B., Patel M., Hampton L.M., Burnett E., Ehlman D.C., Garon J., Cloessner E., Chmielewski E., Hyde T.B., Mantel C. (2017). Administering multiple injectable vaccines during a single visit—Summary of findings from the accelerated introduction of inactivated polio vaccine globally. J. Infect. Dis..

[44] Preza I., Subaiya S., Harris J.B., Ehlman D.C., Wannemuehler K., Wallace A.S., Huseynov S., Hyde T.B., Nelaj E., Bino S. (2017). Acceptance of the administration of multiple injectable vaccines in a single immunization visit in Albania. J. Infect. Dis..

[45] Smith P.J., Humiston S.G., Parnell T., Vannice K.S., Salmon D.A. (2010). The association between intentional delay of vaccine administration and timely childhood vaccination coverage. Public Health Rep..

[46] World Health Organization (2022). Joint Reporting form on Immunization for Ghana Vaccine Supply and Logistics. https://immunizationdata.who.int/.

[47] Nkwenkeu S.F., Jalloh M.F., Walldorf J.A., Zoma R.L., Tarbangdo F., Fall S., Hien S., Combassere R., Ky C., Kambou L. (2020). Health workers’ perceptions and challenges in implementing meningococcal serogroup a conjugate vaccine in the routine childhood immunization schedule in Burkina Faso. BMC Public Health.

[48] Wallace A.S., Krey K., Hustedt J., Burnett E., Choun N., Daniels D., Watkins M.L., Soeung S.C., Duncan R. (2018). Assessment of vaccine wastage rates, missed opportunities, and related knowledge, attitudes and practices during introduction of a second dose of measles-containing vaccine into Cambodia’s national immunization program. Vaccine.

[49] Wolff B., Aborigo R.A., Dalaba M., Opare J.K., Conklin L., Bonsu G., Amponsa-Achiano K. (2023). Community Barriers, Enablers, and Normative Embedding of Second Year of Life Vaccination in Ghana: A Qualitative Study. Glob. Health Sci. Pract..

[50] Smalley H.K., Keskinocak P., Engineer F.G., Pickering L.K. (2011). Universal tool for vaccine scheduling: Applications for children and adults. Interfaces.

[51] Cohen N.J., Lauderdale D.S., Shete P.B., Seal J.B., Daum R.S. (2003). Physician knowledge of catch-up regimens and contraindications for childhood immunizations. Pediatrics.

[52] Engineer F.G., Keskinocak P., Pickering L.K. (2009). OR practice—Catch-up scheduling for childhood vaccination. Oper. Res..

[53] Siddiqi D.A., Ali R.F., Shah M.T., Dharma V.K., Khan A.A., Roy T., Chandir S. (2023). Evaluation of a Mobile-Based Immunization Decision Support System for Scheduling Age-Appropriate Vaccine Schedules for Children Younger Than 2 Years in Pakistan and Bangladesh: Lessons from a Multisite, Mixed Methods Study. JMIR Pediatr. Parent..

[54] Harvey H., Reissland N., Mason J. (2015). Parental reminder, recall and educational interventions to improve early childhood immunisation uptake: A systematic review and meta-analysis. Vaccine.

[55] Oya-Ita A., Nwachukwu C., Oringanje C., Meremikwu M. (2009). Interventions for improving coverage of child immunization in low-income and middle-income countries. Cochrane Database Syst Rev..

[56] Yunusa U., Garba S.N., Umar A.B., Idris S.H., Bello U.L., Abdulrashid I., Mohammed J. (2021). Mobile phone reminders for enhancing uptake, completeness and timeliness of routine childhood immunization in low and middle income countries: A systematic review and meta-analysis. Vaccine.

[57] Aksnes B.N., Walldorf J.A., Nkwenkeu S.F., Zoma R.L., Mirza I., Tarbangdo F., Fall S., Hien S., Ky C., Kambou L. (2021). Vaccination information, motivations, and barriers in the context of meningococcal serogroup A conjugate vaccine introduction: A qualitative assessment among caregivers in Burkina Faso, 2018. Vaccine.

[58] Kulkarni S., Ishizumi A., Eleeza O., Patel P., Feika M., Kamara S., Bangura J., Jalloh U., Koroma M., Sankoh Z. (2023). Using Photovoice Methodology to Uncover Individual-level, Health Systems, and Contextual Barriers to Uptake of Second Dose of Measles Containing Vaccine in Western Area Urban, Sierra Leone, 2020. Vaccine X.

[59] Ansong D., Tawfik D., Williams E., Benson S., Nyanor I., Boakye I., Obirikorang C., Sallah L., Arhin B., Boaheng J.M. (2014). Suboptimal vaccination rates in rural Ghana despite positive caregiver attitudes towards vaccination. J Vaccines Immun..

[60] Rahji F.R., Ndikom C.M. (2013). Factors influencing compliance with immunization regimen among mothers in Ibadan, Nigeria. IOSR J. Nurs. Health Sci..

[61] Brown D.W., Gacic-Dobo M. (2015). Home-based record prevalence among children aged 12–23 months from 180 demographic and health surveys. Vaccine.

[62] Acharya K., Lacoul M., Bietsch K. (2019). Factors Affecting Vaccination Coverage and Retention of Vaccination Cards in Nepal.

[63] Hussain I., Khan A., Rhoda D.A., Ahmed I., Umer M., Ansari U., Shah M.A., Yunus S., Brustrom J., Oelrichs R. (2023). Routine immunization coverage and immunization card retention in Pakistan: Results from a cross-sectional national survey. Pediatr. Infect. Dis. J..

