# Supplementary material for: Timeliness of Childhood Vaccinations Following Strengthening of the Second Year of Life (2YL) Immunization Platform and Introduction of Catch-Up Vaccination Policy in Ghana
Source: Vaccines (Basel). 2024 Jun 27;12(7):716. doi: 10.3390/vaccines12070716 (PMC11281534; doi:10.3390/vaccines12070716)
Supplement: Supplementary file 1 [file vaccines-12-00716-s001.zip › vaccines-3008734-supplementary.pdf]

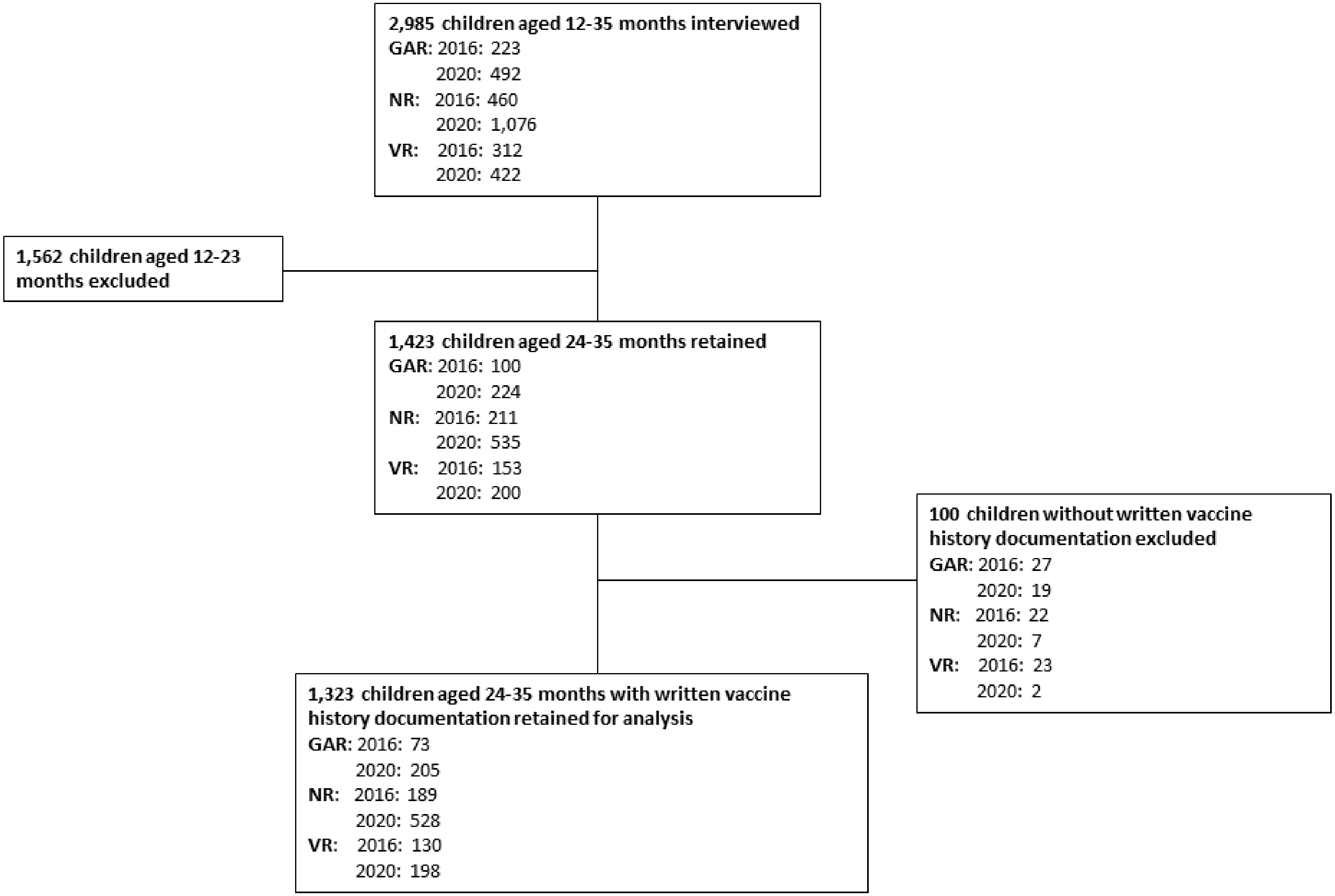

Supplementary figure S1 Flowchart of children included in vaccination timeliness analyses in Greater Accra (GAR), Northern (NR), and Volta (VR) regions during 2016 and 2020.
